# Life-threatening acute water intoxication in a woman undergoing hysteroscopic myomectomy: a case report and review of the literature

**DOI:** 10.1186/s12905-020-0895-y

**Published:** 2020-03-12

**Authors:** Chen-Yi Liao, Chang-Han Lo, Mu-Xian Yu, Wei-Hung Chan, Kuang-yu Wei, Min-Feng Tseng, Chia-Chao Wu

**Affiliations:** 1 Kaohsiung Armed Forces General Hospital, Kaohsiung, Taiwan; 2 Division of Nephrology, Department of Medicine, Tri Service General Hospital, Pen-Hu Branch, Peng-Hu, Taiwan; 3 Division of Nephrology, Department of Internal Medicine, Tri-Service General Hospital, National Defense Medical Center, No. 325, Section 2, Cheng-Kung Road, Neihu, 114 Taipei, Taiwan; 4 Department of Obstetrics and Gynecology, Tri-Service General Hospital, National Defense Medical Center, Taipei, Taiwan

**Keywords:** Acute water intoxication, Cardiopulmonary failure, Hysteroscopic myomectomy, Extracorporeal membrane oxygenation

## Abstract

**Background:**

Acute water intoxication after hysteroscopy is a rare, life-threatening condition, often accompanied with delayed diagnosis owing to masked symptoms because of general anesthesia.

**Case presentation:**

Herein we presented a 39-year-old female who presented with cardiac arrest after hysteroscopic myomectomy because of acute water intoxication and survived after extracorporeal membrane oxygenation, continuous venous–venous hemofiltration, and aggressive high sodium fluid resuscitation.

**Conclusion:**

Failure to recognize and treat this condition appropriately may lead to potentially lethal cardiopulmonary complications.

## Background

Acute water intoxication (AWI, acute hyponatremia) typically occurs when a large quantity of electrolyte-free water is administered to a person with impaired water excretion ability. AWI develops within 48 h and usually manifests as headache, nausea, vomiting, visual disturbances, seizure, coma, and irreversible brain damage. Life-threatening events such as neurogenic stunned myocardium, noncardiogenic pulmonary edema, brain edema, and respiratory arrest can occur [1]. Postoperative AWI is a commonly encountered scenario occurring in 1% of patients because of a combination of hypotonic fluid administration and non-osmotic stimuli. Operations like transurethral resection of the prostate, transcervical resection of the endometrium, transcervical resection of fibroids, and hysteroscopic myomectomy are occasionally associated with postoperative AWI owing to the administration of hypotonic irrigation media. Herein we presented the case of a young woman who suffered a cardiac arrest resulting from AWI during hysteroscopic myomectomy and was successfully treated with extracorporeal membrane oxygenation (ECMO), continuous venous–venous hemofiltration (CVVH), and prompt correction of plasma sodium.

## Case presentation

A 39-year-old healthy woman with normal menstrual periods presented to the emergency department because of postoperative cardiac arrest after a hysteroscopic myomectomy with general anesthesia. Preoperative blood tests revealed no anomalies. According to the local health facility, she had been irrigated with 8000 mL of 5% dextrose water with monopolar electrosurgery and subsequently underwent 35 min of cardiopulmonary resuscitation with medication including sodium bicarbonate 266 mmol (16 ampules) and other inotropic agents. On admission, her body weight was 74 kg (compared to presurgery body weight 62 kg); body mass index of 26.2; equal and dilated pupils; Glasgow coma scale of E1VTM1; low body temperature (32.9 °C), rapid heart rate (117 bpm), hypotension (74/47 mmHg), desaturation with SpO_2_ of 86% and less than 100% FiO_2_ support after intubation, and she was placed on ventilator support with a lung-protective strategy. Her laboratory tests revealed iso-osmotic hyponatremia, severe metabolic acidosis with hypercapnia and hypoxemia, hypoalbuminemia, and hemolysis with disseminated intravascular coagulopathy. The patient received a significant amount of sodium bicarbonate, so the acidosis was most likely much more severe at presentation and the acidosis may have been due to lactic acidosis. (Table 1).

**Table 1:** Biochemical studies on admission

Study	Values	Reference range
Plasma		
PH	7.155 ^a^	7.35–7.45
PCO2, mmHg	50^a^	35.0–45.0
PO2, mmHg	60.1	75–100
HCO3^−^,mmol/L	17.2 ^a^	22.0–27.0
Na^+^, mmol/L	125 ^a^	136–145
Osmolarity, mOsm/kgH2O	277	275–295
K^+^,mmol/L	3.8	3.5–5.1
Cl^−^, mmol/L	98	98–107
Glucose, mg/dL	334	74–100
BUN, mmol/L	13	7–25
Creatinine, mg/d	1.3 ^a^	0.7–1.2
Albumin, g/dL	< 1.5 ^a^	3.5–5.7
Hemoglobin, mg/dL	11.0	12.0–16.0
Haptoglobin, mg/d	7.3	36.0–195.0
LDH, U/L	1352	140–271
Lactic acid mmol/L	10.9	0.5–2.2
Total bilirubin, mg/d	3.5	0.3 to 1.0
Direct bilirubin, mg/d	0.5	< 0.2
PT, sec	28.6	8.0–12.0
aPTT, sec	65.4	23.9–35.5
D-dimer, FEU	> 35.20	< 0.5
Endocrine survey (Before discharge)		
Cortisol,ug/dL	15.25	
ACTH, pg/mL	19.6	0.1–46
TSH,uIU/ml	5.41	0.25–5.00
Free T4,ng/dl	1.29	0.89–1.78

*AST* aspartate aminotransferase; *ALT* alanine aminotransferase; *ACTH* adrenocorticotropic hormone; *TSH* thyroid-stimulating hormone

^a^ indicates abnormal values

**Fig. 1:**
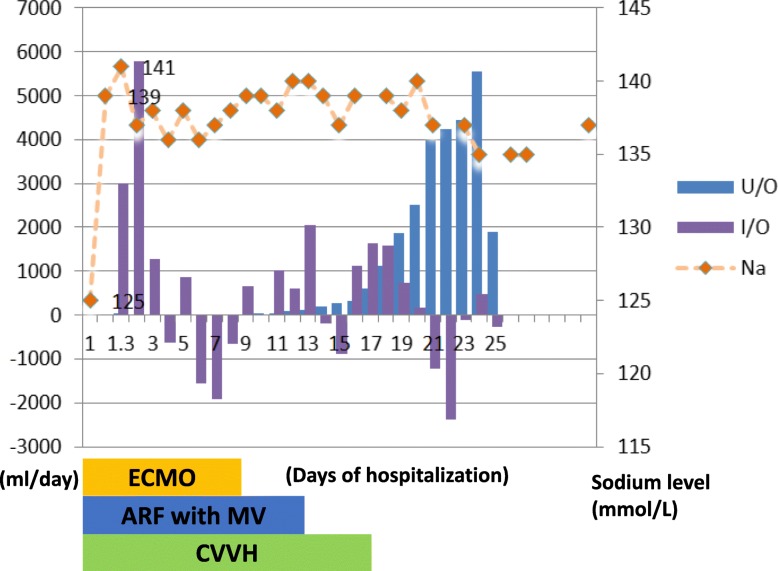
Daily profiles of urine output (U/O), intake and output (I/O), and serum sodium levels (Na) of 25 days of hospitalization. Normal sodium level range from 136 to 145 mmol/L.ADM: admission; ECMO: extracorporeal membrane oxygenation; ARF with MV: acute respiratory failure with mechanical ventilation. CVVH: continuous venovenous hemofiltration . Marked amount of urine produced in the first 14 days showed apparently negative values. Serum sodium level normalized from 125 to 139 mmol/L after resuscitation with 300 ml 3% saline, sodium bicarbonate 242 mEq and CVVH in the first 27 h after admission

The patient was immediately resuscitated with 300 mL of intravenous 3% sodium chloride within 4 h for acute hyponatremia. Because of altered consciousness, mannitol and furosemide were administered for suspected brain edema. Inotropic agents and vasopressors were administered for profound shock status. Initial chest X-ray and chest CT were compatible with acute pulmonary edema (Fig. 1a). Echocardiogram revealed severe hypokinesia of the left ventricular apex with ejection fraction of 35%.Although initial troponin level was 1.944 ng/mL, subsequently, the levels increased to 18.46 and 20.73 ng/mL. After resuscitation, serum sodium level increased from 125 mmol/L to 139 mmol/L 3 h after admission and was 141 mmol/L 7 h after admission; however, the patient remained comatose. Brain CT 1 day after admission revealed no evidence of focal swelling. Subsequently, she was treated with CVVH to remove accumulated fluid from acute pulmonary edema and anuric acute kidney injury, which occurred despite the use of diuretics. Because of acute respiratory distress syndrome with severe hypercapnia, hypoxemia, and poor motion of the heart wall, the patient received venous–arterial ECMO. Further follow-up laboratory examinations revealed unremarkable thyroid function and adrenal function (Table 1). As follow-up echocardiography revealed improved left ventricular diastolic and systolic function with ejection fraction of 57%, the patient was successfully weaned off ECMO on the day 9; she regained consciousness on day 10 and was successfully extubated on the day 13 of admission (Fig. 1). She was relocated to the ward on postoperative day 25 and was released from the hospital on postoperative day 28 without any detectable sequelae.

## Discussion and conclusions

Hysteroscopic procedures are performed using an endoscope, with intrauterine distention using either gas (CO_2_) or fluid distending media. The procedure can lead to life-threatening events if systemic absorption of distending media such as AWI occurs (0.06–0.2% of women) [2].

Excessive absorption of non-electrolyte distending media such as 1.5% glycine (200 mOsm/L), 3% sorbitol (165 mOsm/L), and 5% dextrose can cause AWI [3]. In patients undergoing hysteroscopic procedures, symptoms develop when serum sodium concentration drops below 125 mmol/L [3].

Premenopause, hypoxia, and young age are common risk factors associated with worsened prognosis of hyponatremia encephalopathy in postoperative AWI. The relative risk of death or permanent neurological damage from hyponatremic encephalopathy is 30 times higher for women than men and 25 times greater for menstruating compared to postmenopausal females [4]. The proposed mechanism is associated with the inhibitory effects of estrogen on the ATPase pump that regulates electrolyte flow through the blood–brain barrier [5]. Additionally, the effects of vasopressin on cerebral vasoconstriction and hypoperfusion of brain tissue also precipitate the risk of hyponatremic encephalopathy in premenopausal women [6]. The differential diagnosis of cardiopulmonary failure and acute consciousness disturbance after hysteroscopy in the present case included AWI, acute gas embolism, and thromboembolism. Irrigation media included hypotonic, normal tonic, and gas-like CO_2_. Gas media contribute to a higher risk of developing acute air embolism during hysteroscopy. Although gas media was not administered in the present case, the incidence of acute air embolism is higher in patients undergoing bipolar electrosurgery, especially when more than 1000 mL of fluid is absorbed [3, 6]. In the present case, we believed that the patient suffered from postoperative AWI (lower than 125 mmol/L) before admission to the emergency department because of the large amount of sodium bicarbonate (266 mmol) resuscitation during CPR.

Common risk factors of AWI in patients undergoing hysteroscopic procedures include prolonged operative time, large irrigation fluid amount, higher pressure created by the intrauterine media with higher systemic absorption, visceral perforation, and general anesthesia [3, 6]. The actual volume of distension fluid often exceeds the declared volume by 2.8–10% owing to incomplete collection of spilled fluids or false lower fluid deficit as a result of significant bleeding during hysteroscopic surgery as in the present case [7]. The AAGL Guidelines consensus view is that once a fluid deficit of 1000 ml of hypotonic solution or 2500 ml with an isotonic solution is reached immediate suspension of the procedure is imperative. When high-viscosity distending media are used, the maximum infused volume should not exceed 500 mL, and in the elderly and those with cardiopulmonary compromise should not exceed 300 mL [6]. Patients with decreased serum sodium of 10 meq/L, which is representative of 1000 mL of hypo-osmotic irrigation fluid absorption in women undergoing hysteroscopy, are more are likely to develop neurological symptoms [4, 6]. Thus, we believe that irrigation of 8000 mL of dextrose water with absorption of more than 1000 mL occurred in our patient. Sudden-onset neurogenic stunned myocardium has been reported after AWI especially in a younger woman [1]. Neither irrigation fluid absorption related non-cardiogenic pulmonary edema nor postcardiopulmonary resuscitation pulmonary edema can lead to severe hypoxemia, which is a strong predictor of high mortality in AWI [4].The danger of combined hypoxemia and hyponatremia should be stress out because hypoxemia impairs the ability of the.

brain to adapt to hyponatraemia, leading to a vicious cycle of worsening hyponatraemic encephalopathy. Hyponatraemia cause derangement in both cerebral.

blood flow and arterial oxygen content. Symptomatic hyponatremia can lead to hypoxemia through both non-cardiogenic pulmonary edema and hypercapnic respiratory failure. Besides, the cerebral edema from hyponatremia also lead to non-cardiogenic pulmonary edema [4]. Thus, early recognition of AWI with the aid of ECMO support in cardiopulmonary decompensation is vital. With the presentation of anuric acute kidney injury with severe metabolic acidosis [8], hemodialysis can rapidly correct hyponatremia, acidosis, osmotic derangements, and volume expansion as well as remove non-electrolyte irrigation fluid. In hemodynamically unstable conditions, CVVH is the better choice [9].

Plasma sodium level slowly and continuously shifts toward normal with CVVH treatment, rendering it a safe and effective option for the treatment of acute hyponatremia. Moreover, the rate of sodium correction may be controlled by changing the dialysate or the delivery and composition of the replacement fluid [8]. In the present case, the sodium correction ratewas rapid (16 mmol/L within 7 h), relative to acute symptomatic hyponatremia without developing osmotic demyelination. Currently, no data from controlled trials exist to enable the examination of maximal correction rate in AWI after hysteroscopy. A correction rate of 25 mEq/L is generally recommended within 48 h. The correction rates reported by most publications were higher than that in the present case despite developing osmotic demyelination [10].

The utmost caution is necessary when looking for hidden risk factors prior to hysteroscopic procedures. During surgery, constant vigilance regarding change in vital signs, input, output, irrigation fluid amount, and duration are critical in preventing AWI. Once AWI manifests, rapid elimination of free water deficits and normalization of sodium levels by adopting measures such as hypertonic saline and CRRT in anuric cases, and ECMO therapy in cases of severe cardiopulmonary decompensation is essential.

## Data Availability

Not applicable. The patient’s data is only available from our hospital eletronic database which is not open to the public.

## Abbreviations

AWI: Acute water intoxication
CPR: Cardiopulmonary resuscitation
CT: Computed tomography
CVVH: Contionus venous-venous hemofiltration
ECMO: Extracorporeal membrane oxygenation
